# Complicated Inguinocrural Hernias: Laparoscopic Vs. Open Surgery in the Emergency Setting

**DOI:** 10.3389/jaws.2025.14408

**Published:** 2025-03-11

**Authors:** Lucía Aragone, Nicolás Rosasco, Juana Gutierrez, Raul Croceri, Pablo Medina, Daniel Pirchi

**Affiliations:** Abdominal Wall Unit, General Surgery Department, British Hospital of Buenos Aires, Buenos Aires, Argentina

**Keywords:** complicated inguinocrural hernias, incarcerated hernia, strangulated hernia, laparoscopic hernia repair, conventional inguinocrural hernia repair

## Abstract

**Introduction:**

The feasibility of laparoscopic treatment for inguinocrural-hernias (ICH) and its advantages over open techniques have already been demonstrated. Nonetheless, there is still no sufficient literature regarding laparoscopy for incarcerated or strangulated ICH in the emergency setting. Our primary outcome was to evaluate the feasibility and safety of laparoscopic surgery (LS) for complicated ICH by comparing outcomes to open surgery (OS).

**Methods:**

A comparative retrospective study with prospective case registry was conducted. All patients who underwent ICH repair due to complicated hernias from January 2003 to December 2023 were analyzed and divided into groups according to the approach during surgery: OS (by Lichtenstein technique) or LS (by transabdominal preperitoneal approach). Demographic variables, hernia size and type, surgical time, length of stay, recurrence and other morbidities were compared between groups.

**Results:**

A total of 8282 ICH were operated in the studied period, out of which 162 were included in the study due to incarceration or strangulation. Of these, 83 were treated by OS, while 79 underwent LS. LS showed a reduction in surgical time (70 min IQR60-103 vs. 117 min IQR100-120; p 0.03), length of stay (1.9 days ± 1.4 vs. 2.9 days ± 3.1; p 0.01) and total morbidities (6.3% vs, 16.8%; p 0.04), with a similar recurrence rate (1.2% vs. 1.2%; p1) when compared to OS group.

**Conclusion:**

Laparoscopic surgery for the treatment of complicated inguinocrural-hernias is a feasible and safe approach. It allows the benefits of minimally invasive surgery, including shorter surgical time, shorter length of stay and fewer postoperative morbidities, without increasing recurrence rate compared to open surgery.

## Introduction

Worldwide, over 20 million patients undergo inguinal hernia repair annually [1]. The feasibility of laparoscopic treatment for inguinal hernias and its advantages over open techniques have already been demonstrated [2, 3]. Laparoscopic inguinal hernia repair (LIHR) was associated with earlier discharge from hospital, quicker return to normal activity and work and significantly fewer postoperative complications than open inguinal hernia repair [4]. In experienced centers, LIHR has become the standard of care due to reported benefits such as reduced pain and rapid postoperative recovery [5].

Nonetheless, there is no sufficient literature regarding laparoscopic approach for the treatment of complicated inguinocrural hernias in the emergency setting. Incarcerated or strangulated inguinocrural hernias are a common pathology for general surgeons [6]. These were classically treated by open surgery but with the development of laparoscopy, the question remains if the benefits of this minimally invasive technique could be transferred to patients with complicated inguinocrural hernias. Actual guidelines report that due to the lack of evidence of benefits of one technique over the other, in the case of complicated hernias in the emergency setting an individualized approach is preferred [5].

Our primary outcome was to evaluate the feasibility and safety of the laparoscopic approach for complicated inguinocrural hernias by comparing outcomes to the open surgery approach.

## Methods

### Study Design and Population

A comparative retrospective study with prospective case registry was conducted. All patients who underwent inguinocrural hernia repair due to complicated hernias from January 2003 to December 2023 in a high-volume center were analyzed.

The study included patients with diagnosis of incarcerated or strangulated inguinocrural hernias operated by open or laparoscopic surgery. All patients were assessed by surgeons preoperatively. Diagnosis was made by physical examination, after impossibility of reduction of the herniated bowel. Since physical examination alone is sufficient for diagnosis, no systematic preoperative imaging was performed, to ensure prompt treatment and preserve bowel vitality. Ultrasound or Computerized Tomography Scan were performed only in case of diagnostic doubt.

Patients were divided into groups according to the approach during surgery, open (OS) or laparoscopic (LS). The approach during surgery, open or laparoscopic, was decided according to the surgeon’s criteria, patient by patient and taking into account comorbidities, history of previous abdominal surgeries and physical examination.

In patients operated by open surgery a Lichtenstein technique was chosen. In patients operated by laparoscopy a transabdominal preperitoneal approach (TAPP) approach was chosen.

Patients with diagnosis other than complicated inguinocrural hernias, those patients whose hernia repair was performed with other procedure at the same time, those patients with diagnosis of ventral complicated hernias and those patients with loss of follow-up were excluded. In addition, patients with intraoperative findings of bowel perforation or peritonitis were excluded. In these cases, drainage, lavage, and bowel resection were performed either laparoscopic or by laparotomy, while hernia repair was deferred to a second posterior surgery, as local conditions after peritonitis or perforation were not considered optimal for hernia repair with mesh. Patients with complicated inguinocrural hernias that required conversion from laparoscopic to open surgery were excluded.

### Surgical Technique

All surgeries were performed by the same team of abdominal wall attending surgeons who are highly experienced in laparoscopic surgery, all trained at our center and, to date, have performed more than 11,000 LIHR over the past 25 years.

**FIGURE 1:**
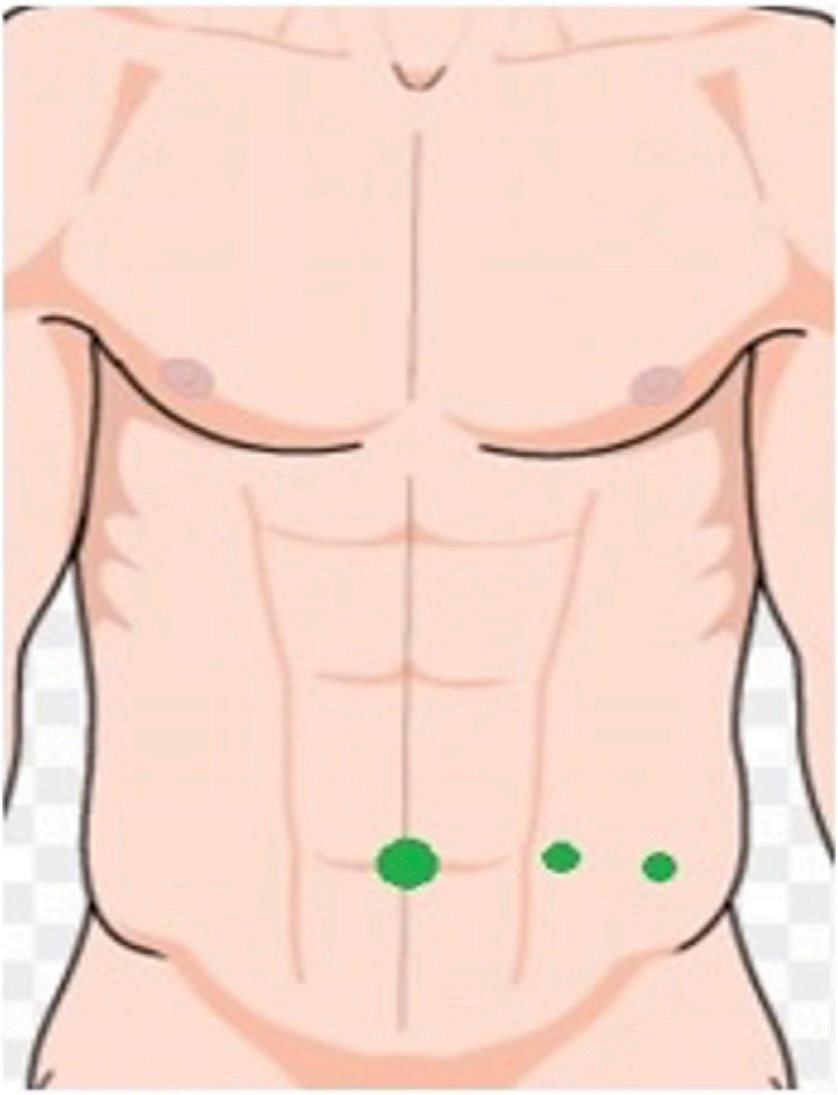
Laparoscopic inguinal hernia repair (LIHR) by transabdominal preperitoneal approach (TAPP) approach. Dots represent 12 mm umbilical port and 2 accessory 5 mm ports on the left flank.

**FIGURE 2:**
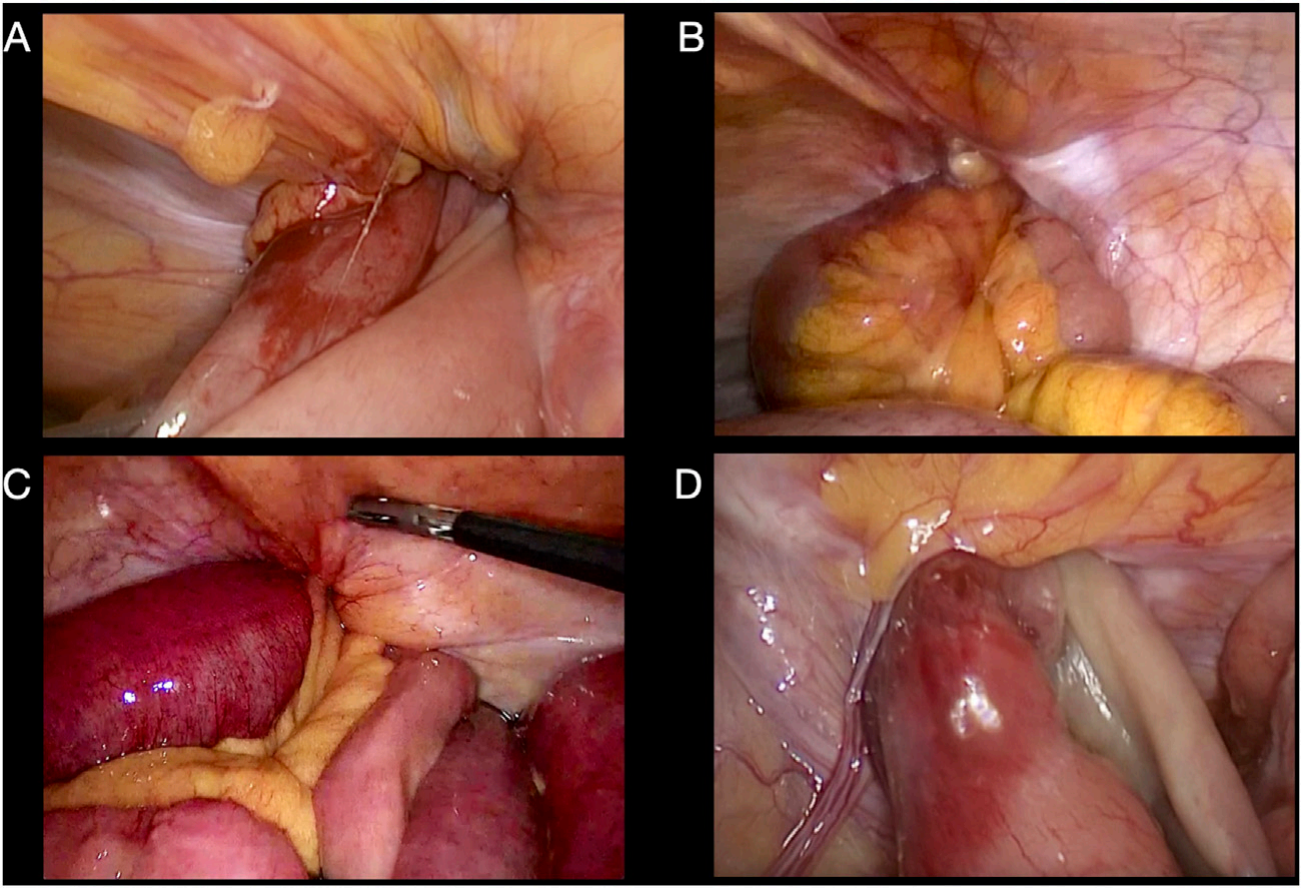
Intraoperative laparoscopic view. **(A, B)** Right-side incarcerated inguinal hernias. **(C, D)** Left-side strangulated inguinal hernias.

TAPP approach was used in all cases operated by laparoscopy. The procedure was performed under general anesthesia, using a 12 mm umbilical port and 2 accessory 5 mm ports on the left flank, independently of the side of the hernia. See Figure 1. Firstly, an exploratory laparoscopy is performed with reduction of the strangulated or incarcerated intestinal content. See Figure 2. Bimanual maneuvers or opening of the hernial ring were performed in some cases, if content reduction was not possible with only intraabdominal maneuvers. After peritoneal flap creation and hernia reduction, polypropylene meshes with absorbable fixation devices were used. Polypropylene meshes were introduced to the abdominal cavity rolled, then unfolded and placed into the previously dissected flaps. All meshes were fixed to the tissue using four absorbable tracks, two at the Cooper’s ligament and two above the iliopubic tract. After mesh placement, flaps were closed by barbed continuous absorbable suture. If the herniated bowel did not recover vitality after the hernia repair and irrigation with warm water, segmentary enterectomy with end-to-end manual suture was performed through umbilical mini-laparotomy with exteriorization of the compromised section of bowel.

Lichtenstein technique with placement of polypropylene mesh was performed in all patients treated by open surgery included in this study. If the herniated bowel did not regain vitality after reduction, segmentary enterectomy was performed through the inguinal conduct, prior to the repair.

### Postoperative Care

All patients were admitted at least for 24 h of postoperative monitoring. If able to control pain, urinate and restore proper oral intake, patients were discharged. Patients were examined as outpatients at 7 days, 1, 6, 12, 18, and 24 months. In addition to in-person follow-up visits, telephone follow-up was completed for those patients who lost the ability to attend in-person follow-ups after 1-month postoperative time. Recurrence was detected through physical examinations and, if necessary, ultrasound.

### Data Collection

Demographic variables, hernia type and size (according to EHS Classification [5]), surgical time (minutes), length of stay (days), recurrence and other morbidities were prospectively recorded and compared between both groups.

### Ethical Approval

This study was reviewed and approved by institutional review board (IRB) and the written informed consent was waived by the IRB owing to the study’s retrospective nature.

### Sample Size Calculation

Sample size was determined taking into account previously published study’s length of stay (days) in emergency laparoscopic or open IHR with 95% confidence interval, 80% power and 1:1 ratio between groups [7]. Sample size obtained after calculation was 39 cases for each group. Therefore, we could include approximately 160 eligible patients with a 1:1 ratio between groups over a period of 20 years.

### Statistical Analysis

Statistical analysis was performed using *Jamovi* Computer Software (Version 2.4.12.0; Sydney, Australia). Descriptive variables are set as mean and standard deviation (SD) or median and interquartile range (IQR) and the qualitative variables as percentages. Comparison of the two groups was performed using the Mann Whitney and Fisher tests, respectively. A p value < 0.05 was considered statistically significant.

## Results

**FIGURE 3:**
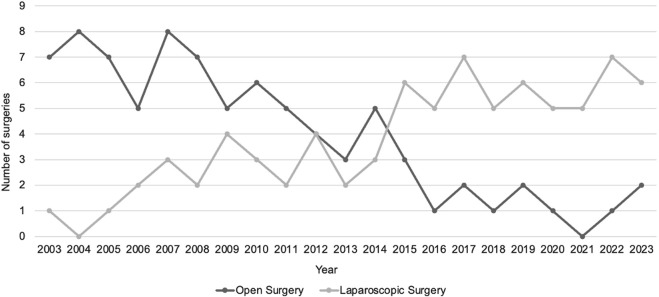
Distribution of patients between groups through the years.

During the study period a total of 8782 inguinocrural hernia repairs were performed, of which 166 were classified as complicated due to incarceration or strangulation. Of these, 83 were treated by OS, while 79 underwent LS. A total of 4 patients were converted from laparoscopic to open surgery and were excluded from this study; causes of conversion included failure to reduce the herniated bowel, lack of space in the abdominal cavity during laparoscopy due to significant bowel distension and abdominal adhesions. The distribution of patients between groups through the years is shown in Figure 3. The mean age of patients was 65.6 years (±15.6), the average body mass index (BMI) was 26.2 kg/m2 (±4.1) and 69.7% of patients (113) were male, with no statistically significant differences between groups. Although a higher percentage of patients with ASA (American Society of Anesthesiologists) Score III/IV and a history of previous abdominal surgeries were approached via OS, no statistically significant differences were found between the groups. Patient’s characteristics are detailed in Table 1.

**TABLE 1:** Demographic/perioperative data.

	Open surgery n = 83	Laparoscopic surgery n = 79	p
Mean age, years (SD)	68.9 (13.7)	61.7 (16.9)	0.2
Mean body mass index, kg/m^2^ (SD)	25.8 (3.5)	26.4 (4.3)	0.6
Male, *n* (%)	56 (67.4)	57 (72.1)	0.5
ASA (American Society of Anesthesiologists) Score			
I/ii, n (%)	52 (62.7)	60 (75.9)	0.08
III/IV, n (%)	31 (37.3)	19 (24.1)	
Previous abdominal surgeries, n (%)	51 (61.4)	38 (48.1)	0.1
Lateral hernias, *n* (%)	62 (74.6)	57 (72.1)	0.7
L1 hernias, *n* (%)	2 (3.2)	1 (1.7)	1
L2 hernias, *n* (%)	33 (53.2)	27 (47.3)	0.5
L3 hernias, *n* (%)	27 (43.5)	29 (50.8)	0.6
Medial hernias, *n* (%)	10 (12.1)	14 (17.7)	0.3
M1 hernias, *n* (%)	0	0	1
M2 hernias, *n* (%)	0	1 (7.1)	0.4
M3 hernias, *n* (%)	10 (100)	13 (92.8)	0.5
Femoral hernias, *n* (%)	11 (13.2)	8 (10.1)	0.6
F1 hernias, *n* (%)	1 (9.1)	0	1
F2 hernias, *n* (%)	3 (27.2)	2 (25)	1
F3 hernias, *n* (%)	7 (63.6)	6 (75)	1
Right hernias, *n* (%)	51 (61.4)	45 (57.9)	0.6
Left hernias, *n* (%)	32 (38.6)	34 (43.1)	0.6
Bowel resection, *n* (%)	11 (13.2)	9 (11.3)	0.8
Mean mesh size, cm^2^ (SD)	102.5 (27.3)	166.6 (35.4)	**0.001**
Median surgical time, minutes (IQR)	117 (100–120)	70 (60–103)	**0.03**

p < 0.05 are denoted in bold.

A total of 19 complicated femoral hernias were treated, accounting for 11.7% of the series. In the OS group 11 patients had femoral hernias, while in the LS group, 8 patients had femoral hernias. Hernia’s characteristics are detailed in Table 1.

Bowel resection was required in 11 patients (13.2%) in OS group and in 9 patients (11.3%) in LS group (p 0.8). The median operative time was 117 min (IQR 100–120) in OS group and 70 min (IQR 60–103) in LS group, showing a statistically significant reduction of surgical time for LS group (p 0.03). See Table 1.

The mean length of stay for the OS group was 2.96 (±3.1) days and 1.9 (±1.4) in LS group with a statistically significant difference between groups (p 0.01). See Table 2.

**TABLE 2:** Postoperative data.

	Open surgery n = 83	Laparoscopic surgery n = 79	p
Mean length of stay, days (SD)	2.9 (3.1)	1.9 (1.4)	**0.01**
Mean follow up time, months (SD)	19.2 (4.7)	20.3 (2.5)	0.1
Morbidities, *n* (%)	14 (16.8)	5 (6.3)	**0.04**
Seroma, *n* (%)	8 (9.6)	4 (5.1)	0.3
Acute urinary retention, *n* (%)	1 (1.2)	0	1
Surgical site infection, *n* (%)	4 (4.8)	1 (1.2)	0.3
Testicular hematoma, *n* (%)	1 (1.2)	0	1
Recurrence, *n* (%)	1 (1.2)	1 (1.2)	1

p < 0.05 are denoted in bold.

The recurrence rate of the series was 0.6% (2 cases), with 1 recurrence in OS group and 1 recurrence in LS group, with a mean follow-up of 19.2 (±4.7) months in OS group and 20.3 (2.5) in LS group. There were 19 (11.7%) morbidities recorded, 14 in the OS group (16.8%) and 5 (6.3%) in the LS group, with a statistically significant difference between groups (p 0.04). See Table 2.

## Discussion

The feasibility of laparoscopic treatment for inguinal hernias and its advantages over open techniques have already been demonstrated [2–4]. However, despite the known benefits of laparoscopy, there is no sufficient literature regarding laparoscopic approach for the treatment of complicated inguinocrural hernias and there is no consensus on management of complicated abdominal hernias [5–8].

Currently, laparoscopy is considered the standard procedure for managing acute abdomen in the emergency settings [9]. In addition to its therapeutic utility, laparoscopy offers diagnostic value by allowing assessment of the entire abdominal cavity, thereby facilitating the exclusion of associated pathologies and differential diagnosis [10].

Since the introduction of laparoscopy to the management of the acute abdomen, it has gained widespread acceptance due to several advantages, including: a comprehensive 360° evaluation of the abdominal cavity, the ability to identify other pathologies, pneumoperitoneum´s contribution to the reduction of herniated bowel, sometimes even allowing for spontaneous reduction due to carbon dioxide pressure, the opportunity to perform hernia repair while simultaneously assessing visceral involvement and a thorough evaluation of the need for bowel resection and its extent [11–13].

Conventional open surgery is still recommended for patients with contraindications to laparoscopic surgery or in centers with limited experience in laparoscopy [14]. However, one of its drawbacks is that, in cases with visceral involvement, bowel resection and anastomosis must be performed before the hernia repair, potentially increasing the rate of segmental bowel resections. Laparoscopic surgery offers all the benefits of a minimally invasive approach, including the potential to monitor the affected bowel while performing the repair, allowing time for potential recovery without the need for resection [11]. This approach enables a complete evaluation of the abdominal cavity, favors the reduction of herniated contents through pneumoperitoneum, and reduces the rate and extent of bowel resections [15].

Actual guidelines report that due to the lack of evidence of benefits of one technique over the other, in the case of complicated hernias in the emergency setting an individualized approach is preferred [5].

In our retrospective study, we can see how through the years, the percentage of patients treated by LS increased year by year (Figure 3), showing how the surgical team gained confidence and skill in the management of complicated inguinocrural hernias by laparoscopy. To date, patients with a history of severe respiratory conditions, previous abdominal surgeries, or significant abdominal distension are eligible for a preferred OS approach. These conditions hinder laparoscopic surgery due to pneumoperitoneum intolerance, difficulty accessing the abdominal cavity, and reduced intra-abdominal space during laparoscopy, respectively.

In our series, by comparing the LS and OS group, the benefits of laparoscopy were demonstrated, showing a reduction in surgical time (p 0.03), length of stay (p 0.01) and total morbidities (p 0.04), with a similar recurrence rate (p 1).

### Strengths and Limitations

Our study’s strength lies on it taking place in a high-volume center with a depurated surgical technique performed by the same group of highly trained abdominal wall surgeons. However, the main limitation of this study is its retrospective, single-center design. We also recognize the results can be affected by bias (mainly selection and performance bias).

### Conclusion

Laparoscopic surgery for the treatment of complicated inguinocrural hernias is a feasible and safe approach. It allows the benefits of minimally invasive surgery, including shorter surgical time, shorter length of stay and fewer postoperative morbidities, without increasing recurrence rate compared to open surgery. Further prospective randomized studies are needed to confirm these findings.

## Data Availability

The original contributions presented in the study are included in the article/supplementary material, further inquiries can be directed to the corresponding author.
